# Assessing Quality of Life in Older Adult Patients with Skin Disorders

**DOI:** 10.5539/gjhs.v4n2p119

**Published:** 2012-03-01

**Authors:** Miranda A. Farage, Kenneth W. Miller, Susan N. Sherman, Joel Tsevat

**Affiliations:** The Procter & Gamble Company, 6110 Center Hill Ave Box 136, Cincinnati, Ohio, 45224, USA Tel: 513-634-5594 E-mail: farage.m@pg.com; The Procter & Gamble Company, 6110 Center Hill Ave Box 136, Cincinnati, Ohio, 45224, USA Tel: 513-634-5594 E-mail: farage.m@pg.com; SNS Research, Cincinnati, Ohio, USA; Section of Outcomes Research, Division of General Internal Medicine Department of Internal Medicine, University of Cincinnati College of Medicine, Cincinnati, Ohio, USA & Health Services Research & Development Service Veterans Affairs Medical Center, Cincinnati, Ohio, USA

**Keywords:** Quality of life, Dermatology, Skin diseases, Instruments

## Abstract

**Significance for Public Health:**

The global population is aging. In the industrial world, adults over 65 outnumber children and comprise almost 20% of the population in some countries. Older adults experience a number of skin diseases and disorders that substantially affect their quality of life. Opportunity exists for developing and validating health-related quality of life (HRQoL) measures specifically for dermatological conditions most pertinent to older patients.

Older adults experience a number of skin diseases and disorders that substantially affect quality of life. In the last two decades, a number of instruments have been developed for use among general dermatology patients to assess the effects of treatment and disease progression, perceptions of well-being, and the value that patients place on their dermatologic state of health. This chapter reviews some health-related quality of life (HRQoL) (HRQoL) measures developed and validated specifically for dermatological conditions. However, opportunity exists for developing and validating HRQoL measures specifically for dermatological conditions most pertinent to older patients.

## 1. Introduction

**Figure 1:**
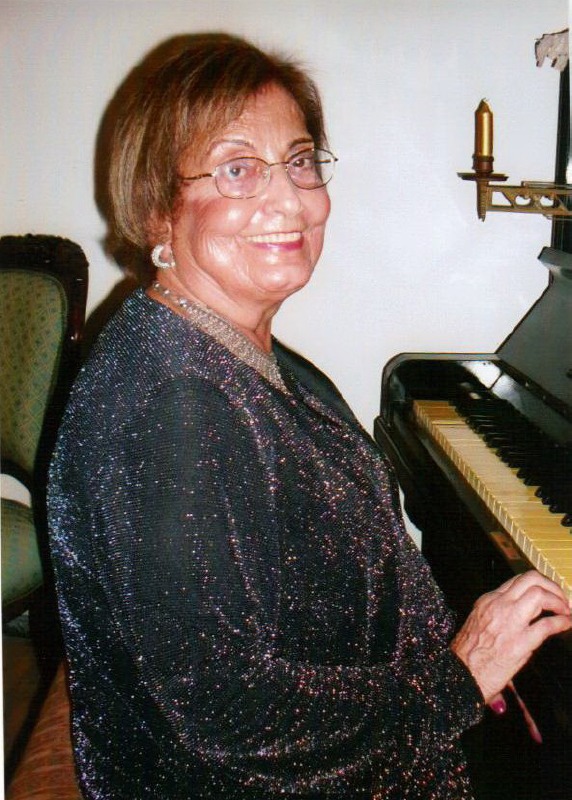
A beautiful lady in her golden years still enjoying playing music on the piano

Measuring HRQoL is an important part of overall patient care Figure 1. Clinical examination and diagnostic testing provide information about patients’ health and the progression (or regression) of disease. An individual’s perception of his or her quality of life is influenced by the person’s physical health, psychological state, level of independence, social relationships, personal beliefs, and relationship to his or her environment. HRQoL assessments allow patients to express their opinions about the value they place on health and how their illness and its treatment affect quality of life. For patients with chronic illness, HRQoL assessment measures changes in the patient’s well-being throughout the course of the disease (Chwalow A.J., 1995). The purpose of this paper is to review HRQoL assessment in general and in skin disease, and to discuss issues related particularly to HRQoL in aging skin.

Quality of life is a simple yet complex paradigm, with philosophers, sociologists, psychologists, economists, theologians, clinicians, and lay persons all having different conceptualizations (Wilson I.B. & Cleary, P.D., 1995; Johnson R.J., & Wolilnsky F.D., 1993; Calman K.C., 1984; Michalos A.C., 1985). While consensus exists that HRQoL is important to patient care, there is no absolute agreement among researchers on how to assess either HRQoL or quality of life in general (Tsevat J. *et al*., 1994; Patrick D.L. & Erickson P., 1993). Nevertheless, two fundamentally different approaches are commonly applied: (1) health status measurement and (2) utility/value/preference assessment.

Health status measures assess various domains of a person’s physical, mental functioning. Health status measures can be either generic (applicable to any disease or health state) or disease-specific (applicable to a single condition or disease) (Tsevat J. *et al*., 1994). One of the most commonly used generic health status instruments is the SF-12 Health Survey, a 12-item measure encompassing eight domains – physical functioning, social functioning, mental health, role limitations due to physical problems, role limitations due to emotional problems, vitality (energy and fatigue), pain, and general health perceptions – each of which is scored separately from 0 (worst) to 100 (best) (Ware J. Jr., Kosinski M. & Keller S.D., 1996). Besides such generic measures, a wide variety of disease-specific measures have been developed. In dermatology, both skin-specific measures (generic dermatology) and skin-disease-specific (e.g., for psoriasis) measures exist. and are reviewed below. Health status measures can be used (1) to interpret and monitor outcomes in clinical treatment programs, (2) as endpoints in clinical trials, (3) to monitor population health, and (4) to estimate the burden of different disease conditions.

Utility/value/preference measures, in contrast to health status measures, assess the value or desirability of a state of health against an external metric such as risk, time, or money (Tsevat J. *et al*., 1994; Torrance G.W., 1986; McCombs K., & Chen S.C., 2007). The most common instruments used to directly measure utility/value/preference, hereafter referred to as utility measures, are (1) the standard gamble, (2) time trade-off, and (3) the rating scale. The standard gamble determines the risk of (usually) death that one would be willing to take to improve a state of health. Scores on the standard gamble can range from 0 to 1, where 0.0 usually represents dead and 1.0 excellent or perfect health. The time-trade-off technique asks how many months or years of life one would be willing to give up in exchange for a better health state. The rating scale (though not a strict measure of utility because it doesn’t involve comparison against an external metric) asks the subject to rate his or her health on a scale, e.g., from 0 to 100, where 0 usually represents dead and 100 perfect health. Utility measures are used primarily to calculate quality-adjusted life years in decision analyses and cost-effectiveness analyses. Whereas health status measures are generally ascertained directly (either from patients, their surrogate decision makers, or their healthcare providers), utility/value/preference measures can be ascertained either directly (from patients, surrogates, providers) or indirectly (by surveying the general population). The indirect assessment of utility involves first assessing the patient’s health status, then mapping a utility derived previously from the general population to that particular state of health. Examples of indirect utility measures include the EQ-5D (Rabin R., & de Charro F., 2001), the SF-6D (Brazier J., Roberts J. & Deverill M., 2002), the Health Utilities Index (Furlong W. *et al.*, 2001), and the Quality of Well-Being Scale (Kaplan R., Anderson J.P., & Ganiats T.G., 1993). A less common utility measure known as willingness to pay assesses the amount of money, either in the form of cash or insurance premiums, one is willing to pay for a cure (Khanna D. *et al*., 2008). This technique is particularly germane to non-life-threatening conditions and, hence, can be applied to many dermatologic diseases.

HRQoL instruments can generally be validated for use in particular populations or for use in a wide variety of cultures, regions and languages (globally-validated instruments or measures). Constructing and validating a new HRQoL instrument entails both qualitative and quantitative methods (Chwalow A.J., 1995). The validity of a questionnaire refers to whether the questionnaire or survey measures what it intends to measure and can be assessed with regard to (1) content, (2) criterion, and (3) construct. Content validity refers to the extent to which a measure (item[s]) adequately describes the underlying statement or question. Content validity can be determined by literature review, expert reviews, or in-depth cognitive interviews. Criterion validity refers to the extent to which survey items predict or agree with an objective assessment of the particular criterion (e.g., in clinical evaluations, patient reports are compared with physician reports or medical records). Construct validity examines the assumption that items measured in the questionnaire correlate with other items hypothesized to measure the same concept (e.g., whether two indicators of emotional well-being agree) and, conversely, whether items or measures in one domain of assessment that are hypothesized to be unrelated to items or measures in another domain of assessment in fact do not correlate (discriminant validity). Correlational and factor analysis are quantitative techniques to assess whether variables are measuring the same underlying construct (Aday L.A., 1996).

The reliability of a questionnaire refers to its reproducibility, that is, the consistency of answers given by the same individual to the same item. Several types of reliability can be assessed. Test-retest reliability examines the correspondence between answers given by an individual when the item(s) is re-administered over a brief interval (sufficiently brief so that an underlying change in health status is not anticipated to occur between administrations). Inter-rater reliability assesses how well data obtained by different interviewers correlate. Internal consistency reliability (as measured by Cronbach’s alpha) assesses how well items within a measure “hang together.”

## 2. Health-Related Quality of Life Measures in Dermatology

As mentioned above, HRQoL measures, in particular health status measures, can be generic or disease-specific. With regards to dermatology, disease-specific measures can be categorized further into instruments for dermatologic conditions in general (skin-specific) or instruments for specific dermatologic conditions (skin-disease-specific, e.g., psoriasis). A review of the literature several years ago identified and critiqued 6 generic health status instruments that have been applied in dermatology, 11 skin-specific measures for adults, and 15 skin-disease-specific measures for use in adults. Herein, we review some of the most commonly used among those (Table 2).

**Table 1:** Age-related changes in skin (http://www.merck.com/mkgr/mmg/sec15/ch122/ch122c.jsp)

Physiologic Decrement	Clinical Consequence(s)
Barrier function	Dryness, ease of irritation
Cell replacement	Rough surface, delayed healing
DNA repair	Increased photocarcinogenesis
Elasticity	Lax skin
Immunologic responsiveness	Chronic low-grade skin infections
Inflammatory responsiveness	Inapparent injuries and infections
Mechanical protection Sensory perception	Frequent injuries Frequent injuries
Sweating	Tendency for hypothermia
Thermoregulation (vascular)	Vulnerability to heat and cold
Vitamin D production	Suboptimal vitamin D stores, osteomalacia, muscle weakness
Wound healing	Persistent wounds, weak scars

**Table 2:** Dermatology-specific instruments for adults*

Disease(s)	Measure	Abbreviation	Author(s)
**Generic for dermatology**			
	Dermatology Life Quality Index	DLQI	Finlay, A.Y.
	Dermatology Quality of Life Scales	DQoLS	Morgan, M.
	Dermatology-Specific Quality of Life Instrument for Contact Dermatitis	DSQL-CD	Anderson, R.T., *<italic>et al</italic>*.
	Family Dermatology Life Quality Index	FDLQI	Basra, M.K., *<italic>et al</italic>*.
	Skindex	Skindex	Chren, M.M., *<italic>et al</italic>*.
**Acne**			
	Acne Disability Index	ADI	Motley, R.J., *<italic>et al</italic>*.
	Dermatology-Specific Quality of Life Instrument for Acne	DSQL-Acne	Anderson, R.T., *<italic>et al</italic>*.
**Alopecia**			
	Kingsley Alopecia Profile	KPA	Kingsley, D.H.
**Atopic dermatitis**			
	Quality of Life Index for Atopic Dermatitis	QoLIAD	Whalley, D., *<italic>et al</italic>*.
**Eczema**			
	Dermatitis Family Impact Questionnaire	DFI	Finlay, A.Y., *<italic>et al</italic>*.
	Patient-Oriented Eczema Measure	POEM	Charman, C.R., *<italic>et al</italic>*.
	Eczema Area and Severity Index	EASI	Hanifin, J.M., *<italic>et al</italic>*.
**Leg ulcer**			
	Charing Cross Venous Ulcer Questionnaire	CCVUQ	Smith, J.J., *<italic>et al</italic>*.
	Diabetic Foot Ulcer Scale	DFS	Bann, C.M., *<italic>et al</italic>*.
	Leg and Foot Ulcer Questionnaire	LFUQ	Hyland, M.E.
**Onychomycosis**			
	Onychomycosis Quality of Life Questionnaire	ONYCHO	Drake, L.A., *<italic>et al</italic>*.
**Psoriasis**			
	Psoriasis Disability Index	PDI	Finlay, A.Y., *<italic>et al</italic>*.
	12-Item Psoriasis Quality of Life Questionnaire	PQOL-12	Koo, J., *<italic>et al</italic>*.
	Psoriatic Arthritis Quality of Life Instrument	PSAQoL	McKenna, S.P., *<italic>et al</italic>*.
**Systemic lupus erythematosus**			
	Systemic Lupus Erythematosus Quality of Life Questionnaire	SLEQoL	Doward, L.C., *<italic>et al</italic>*.

*Adapted from reference

### 2.1 Instruments for Dermatologic Conditions in General

#### 2.1.1 Skindex

Skindex (Chren, M.M. *et al.*, 1996, 1997 & 2001) is a widely used HRQoL instrument applicable to various skin diseases. Skindex originated as a 61-item instrument (Chren M.M. *et al.*, 1996), but over time, it has been reviewed and refined by its developers and today is also available in three shorter versions, a 29-item (Chren, M.M. *et al.*, 1997 & 2001), a 17-item, and a16-item questionnaire (Chren, M.M. *et al.*, 2001). Skindex-61 was developed in the mid-1990s (Chren M.M. *et al.*, 1996). The original version focused on skin diseases and the frequency and severity of their impact on quality of life: cognitive effects, social effects, depression, fear, embarrassment, anger, physical discomfort, and physical limitations. For each domain, patients respond on a scale ranging from 0 (no effect) to 100 (maximum effect). In addition to scores in individual domains, the overall summary score is calculated as the average of responses to items on each domain. When tested in 201 patients, mean (SD) scale scores ranged from 14 (17) for physical limitations to 31 (22) for physical discomfort. Retesting at 72 hours showed the scores to be reproducible and internally consistent. Construct validity (correlation among items thought to measure the same or different concepts) was demonstrated by finding that scores among patients with inflammatory dermatoses were higher (worse) than among patients with isolated lesions, and by exploratory factor analysis (a statistical approach for examining the internal reliability of a measure). Physicians’ opinions of the severity of disease did not correlate consistently with the patients’ Skindex scores, thus indicating that the Skindex instrument may be a useful adjunct to clinical assessment by physicians.

To improve the original Skindex instrument, the original 61-item questionnaire (201 patients) and a revised 29-item questionnaire (Skindex 29) (692 patients) were compared to evaluate the reproducibility, internal consistency reliability, and validity of the new version of the instrument (Chren M.M. *et al.*, 1997). Time needed to complete the questionnaire, the number of items that elicited the same response, and the number of scales that were responsive to patients’ self-reports of changes in their condition were evaluated, as well as reproducibility and construct validity. The questionnaire was completed in 5 minutes on average relative to 15 minutes for the original questionnaire. Only three items elicited the same response from 70% or more of those surveyed, compared with 17 items in the original questionnaire. The revised version was reproducible in a retest at 72 hours and was internally reliable. The 29-item instrument improved upon the original questionnaire in its discriminative and evaluative capability and in administration time (Chren M.M. *et al.*, 2001). Skindex-29 has since been translated into nine languages. Subsequently, the Skindex-29 questionnaire was reduced to a single-page instrument (Skindex-16) assessing how distressed patients were by the disease, as opposed to how frequently they experienced effects of the disease (Chren, M.M. *et al.*, 2001). Skindex-16 has subscales assessing distress related to symptoms, emotions, and functioning (Table 3) and has been translated into 11 languages. Skindex-17, the newest of the Skindex measures, has a psychosocial and a symptom scale. Among all of the dermatology-specific measures reviewed by Both and colleagues, the Skindex-29 was selected as the preferred measure, based on conceptual and measurement model, reliability, validity, responsiveness, item functioning, meaning of scores, administrative burden, respondent burden, availability of alternative forms, and availability of cultural and language adaptations.

**Table 3:** Skindex-16 (Chren, M.M. *et al*., 2001)

	During the past week, how often have you been bothered by:	Never Bothered ↓					Always Bothered ↓	
**1.**	Your skin condition **itching**	◻_0_	◻_1_	◻_2_	◻_3_	◻_4_	◻_5_	◻_6_
**2.**	Your skin condition **burning** or **stinging**	◻_0_	◻_1_	◻_2_	◻_3_	◻_4_	◻_5_	◻_6_
**3.**	Your skin condition **hurting**	◻_0_	◻_1_	◻_2_	◻_3_	◻_4_	◻_5_	◻_6_
**4.**	Your skin condition **being** **irritated**	◻_0_	◻_1_	◻_2_	◻_3_	◻_4_	◻_5_	◻_6_
**5.**	The **persistence / reoccurrence** of your skin condition	◻_0_	◻_1_	◻_2_	◻_3_	◻_4_	◻_5_	◻_6_
**6.**	**Worry** about your skin condition (For example: that it will spread, get worse, scar, be unpredictable, etc)	◻_0_	◻_1_	◻_2_	◻_3_	◻_4_	◻_5_	◻_6_
**7.**	The **appearance** of your skin condition	◻_0_	◻_1_	◻_2_	◻_3_	◻_4_	◻_5_	◻_6_
**8.**	**Frustration** about your skin condition	◻_0_	◻_1_	◻_2_	◻_3_	◻_4_	◻_5_	◻_6_
**9.**	**Embarrassment** about your skin condition	◻_0_	◻_1_	◻_2_	◻_3_	◻_4_	◻_5_	◻_6_
**10.**	**Being annoyed** about your skin condition	◻_0_	◻_1_	◻_2_	◻_3_	◻_4_	◻_5_	◻_6_
**11.**	**Feeling depressed** about your skin condition	◻_0_	◻_1_	◻_2_	◻_3_	◻_4_	◻_5_	◻_6_
**12.**	The effects of your skin condition on your **interactions** **with others** (For example: interactions with family, friends, close relationships, etc)	◻_0_	◻_1_	◻_2_	◻_3_	◻_4_	◻_5_	◻_6_
**13.**	The effects of your skin condition on your **desire to be with people**	◻_0_	◻_1_	◻_2_	◻_3_	◻_4_	◻_5_	◻_6_
**14.**	Your skin condition making it hard to **show affection**	◻_0_	◻_1_	◻_2_	◻_3_	◻_4_	◻_5_	◻_6_
**15.**	The effects of your skin condition on your **daily activities**	◻_0_	◻_1_	◻_2_	◻_3_	◻_4_	◻_5_	◻_6_
**16.**	Your skin condition making it hard to **work or what you do**	◻_0_	◻_1_	◻_2_	◻_3_	◻_4_	◻_5_	◻_6_

Reprinted with permission from Chren, M.M., et al. (2001). Measurement properties of Skindex-16: a brief quality-of-life measure for patients with skin diseases. Journal of Cutaneous Medicine and Surgery, 5, 105-110. Skin-Index 16 is copyrighted and used with kind permission from both Dr. Chren and Springer Publishing Co

**Table:** Skindex-16 scoring

SCALE	ITEMS
Symptoms	1-4
Emotion	5-11
Functioning	12-16

Item scores transformed to 0-100 scale. Scale Score: Average of items in given scale. Total Score: Average of all 16 items

#### 2.1.2 Dermatology Life Quality Index

The Dermatology Life Quality Index (DLQI) is a simple, 10-question, validated questionnaire used widely in clinical settings and available in more than 40 languages (Finlay A.Y. & Khan G.K., 1994). The DLQI was developed based on the responses of 120 patients with various skin diseases who were asked how their disease and its treatment affected their life. For further validation, the DLQI was subsequently administered to 200 consecutive new patients attending a dermatology clinic. Analysis of the responses revealed that atopic eczema, psoriasis, and generalized pruritus have a greater impact on HRQoL than do acne, basal cell carcinoma, and viral warts. When the instrument was administered to 100 healthy volunteers, mean scores were very low. A 1-week, test-retest reliability analysis in 53 patients found that the DLQI instrument was highly reliable (Finlay A.Y. & Khan G.K., 1994).

#### 2.1.3 Dermatology-Specific Quality of Life

The Dermatology-Specific Quality of Life (DSQL) instrument was created to quantify the effect of skin disease on physical discomfort and symptoms, psychological well-being, social functioning, self-care activities, performance at work or school, and self-perceptions (Anderson R.T. & Rajagopalan R., 1997). Reliability and validity were assessed in patients with contact dermatitis or acne vulgaris. The validity of the instrument was assessed by correlating DSQL scores with global ratings of bothersome symptoms and their perceived severity and by the instrument’s ability to discriminate among clinically-defined severity-of-illness groups. Test-retest reliability was assessed at 3 and 7 days. The instrument’s domains had good internal consistency and test-retest reliability. The subscale scores were also moderately-to-highly correlated with globally-validated ratings of symptoms of distress and with overall disease severity. As expected, patients with severe contact dermatitis or scarring from acne vulgaris had higher (worse) DSQL scores than those with milder disease.

#### 2.1.4 The Family Dermatology Life Quality Index

Although skin diseases clearly affect the well-being of patients, instruments that quantify the impact of skin diseases on patients’ family members were lacking. The Family Dermatology Life Quality Index (FDLQI) is a 10-item questionnaire administered to patients’ family members, to measure the indirect impact of skin disease on the family (Basra M.K., Sue H.O.R. & Finlay A.Y., 2007). The FDLQI is responsive to changes in the patient: family members’ scores changed in association with improvement or worsening of the patient’s condition. FDLQI scores and the patients’ DLQI scores, FDLQI scores and inflammatory versus no inflammatory disease, FDLQI scores and the severity of the patient’s disease were strongly statistically associated. There was a positive relationship between FDLQI scores of the family members and the patient’s disease severity, as measured by the DLQI. Thus, the FDLQI has been shown to be simple and practical and a potential additional outcome measure in clinical practice and evaluation research.

#### 2.1.5 The Impact of Chronic Skin Disease on Daily Life

The Impact of Chronic Skin Disease on Daily Life (ISDL) instrument assesses the effect of chronic skin diseases and their treatments on both dermatology-specific and generic aspects of HRQoL (Evers A.W. *et al.*, 2008). The dermatology-specific questions in the ISDL assess physical functioning, itching/scratching, pain, fatigue, and stigmatization; the generic questions assess psychological functioning, illness cognitions, and social support. The reliability and validity of the instrument was assessed in patients with psoriasis or atopic dermatitis. The ISDL is highly reliable, valid, and responsive to changes in health status resulting from ultraviolet B radiation therapy or cognitive behavioral therapy for itching.

#### 2.1.6 Farage Quality of Life Questionnaire

Despite the existence of numerous HRQoL measures, few if any are geared to evaluating the impact of consumer products on HRQoL. A general measure, the Farage Quality of Life Questionnaire (FQoL) was developed to assess the impact of consumer products on various aspects of HRQoL (Farage M. A. *et al.*, 2010). As a prelude to testing the impact on HRQoL of different products, several modules could be developed from the FQoL to capture HRQoL of specific product categories. The self-administered FQoL consists of 27 general items scored on a Likert scale and covering overall quality of life (1 item), well-being (12 items), and energy and vitality (14 items) to assess the potential impact of consumer products on HRQoL. The Well-Being domain has 3 subscales: Emotion, Self-Image, and Self-Competence; the Energy and Vitality domain also has 3 subscales: Personal Pleasure, Physical State, and Routine Activity.

### 2.2 Instruments for Skin-Disease-Specific HRQoL Assesssment

#### 2.2.1 Itch Severity Scale

The Itch Severity Scale (ISS) is a self-administered questionnaire to measure the severity of pruritus (Majeski C.J. *et al.*, 2007). To develop the instrument, an existing pruritus instrument was modified and administered to patients with psoriasis-associated pruritus, along with the RAND-36 Health Status Inventory (Hays R.D. & Morales L.S., 2001) (a generic health status measure) and the DLQI. The resulting ISS contained just seven questions. The ISS is valid and reliable for assessing the severity of pruritus as well as the effectiveness of treatments for pruritus (Majeski C.J. *et al.*, 2007).

#### 2.2.2 Itchy QoL

ItchyQoL is another pruritus-specific HRQoL instrument, consisting of 27 items addressing frequency or bother of symptoms; functional limitations; and emotions (Desai N.S., *et al.*, 2008). Although the authors cited lack of generalizability and potential selection bias as possible limitations, they found this initial pruritus-specific questionnaire to be reliable, valid, and responsive (Desai N.S., *et al.*, 2008).

#### 2.2.3 Scalpdex

Scalpdex (Chen S.C., Yeung J., & Chren M.M., 2002) was the first dermatitis-specific HRQoL instrument developed. Scalpdex captuers three major domains of quality of life – symptoms, functioning, and emotions. The 23-question instrument is reliable, valid, and responsive. Scalpdex can be used by physicians to determine what bothers the patient, as well as to assess the impact of treatments (Chen S.C., Yeung J. & Chren M.M., 2002).

#### 2.2.4 Quality of Life Index for Atopic Dermatitis

Atopic dermatitis is a chronic inflammatory skin condition. The Quality of Life Index for Atopic Dermatitis (QoLIAD) is the first dermatology-specific instrument to assess quality of life impact of atopic dermatitis in adults (Whalley D. *et al.*, 2004). QoLIAD was initially developed based on 65 interviews with patients with atopic dermatitis in the UK, Italy, and the Netherlands. Subsequent field tests involving 993 patients in six different countries resulted in a final 25-item instrument. Psychometric analyses demonstrate the QoLIAD to be a practical, reliable, and valid instrument for assessing the effects of atopic dermatitis and its treatment in clinical practice and in clinical trials (Whalley D. *et al.*, 2004).

#### 2.2.5 Rosacea-Specific Quality of Life Instrument

Acne rosacea (commonly referred to as “rosacea”) is another chronic dermatologic condition that affects up to 10% of the general population (Wilson I.B. & Cleary P.D., 1995; Johnson R.J. & Wolilnsky F.D., 1993; Calman K.C., 1984). The 21-item Rosacea-Specific Quality of Life (RosaQol) instrument (Nicholson K. *et al.*, 2007) addresses symptoms, emotions, and functioning associated with rosacea.

#### 2.2.6 Utility/Value/Preference Measures

Several studies have assessed utilities for one or more skin conditions (McCombs K., & Chen S.C., 2007; Littenberg B. *et al.*, 2003; Motley R.J., & Finlay A.Y., 1989; Finlay A.Y., & Coles E.C., 1995; Chen S.C. *et al.*, 2004; Schmitt J. *et al.*, 2008; Lundberg L. *et al.*, 1999; Chen S., Saheen A., & Garber A., 1998; Zug K.A. *et al*., 1995; Schiffner R. *et al.*, 2002 & 2003; Pitt M., Garsido R. & Stein K., 2006; Williamson D., Gonzales M. & Finlay A. Y., 2001). Perceived utility or value varies not only from condition to condition, but also from patient to patient with a given condition, by assessment method, and by respondent type (McCombs K., & Chen S.C., 2007; Schmitt J. *et al.*, 2008; Lundberg L. *et al.*, 1999; Chen S., Saheen A., & Garber A., 1998; Zug K.A. *et al*., 1995). For example, utility measures for controlled atopic eczema, uncontrolled atopic eczema, controlled psoriasis, and uncontrolled psoriasis have been evaluated in the general population in Germany and in German patients with atopic eczema or psoriasis (Schmitt J. *et al.*, 2008). For the time-trade-off measure of utility, the median score for controlled atopic eczema was 0.97, indicating that respondents were willing to give up a median of 3% of their life expectancy (= [1.0-0.97] × 100%) in order to have perfect health (no atopic eczema). For controlled psoriasis, the median time-trade-off utility measure was 0.93. By contrast, median utility measures were much lower for uncontrolled atopic eczema (0.64) and uncontrolled psoriasis (0.56). The study also asked people about willingness-to-pay. People from the general population would be willing to pay a median of 50€/month for an effective treatment (with no side effects) for controlled atopic eczema, 150€/month for uncontrolled atopic eczema, 75€/month for controlled psoriasis, and 200€/month for uncontrolled psoriasis. Another study reported that patients with psoriasis would be willing to pay on average 14% of their monthly income to be rid of their psoriasis (Schiffner R. *et al.*, 2003).

Several researchers have modified the time-trade-off method by asking patients how many hours each day they would be willing to devote to treating their skin condition if the treatment were curative (Finlay A.Y. & Coles E.C., 1995; Schiffner R., *et al.*, 2002 & 2003). One study indicated that patients with psoriasis would be willing to spend a mean (SD) of 2.8 (3.7) hours a day to be relieved of their psoriasis (Schiffner R., *et al.*, 2003) and 1.2 (0.9) hours a day to be rid of their port wine stains (Schiffner R., *et al.*, 2002).

In a particularly comprehensive study of utility measures for dermatologic conditions, time-trade-off measures of utility were collected for each skin condition experienced by 236 patients (Chen S.C., Bayoumi A.M., Soon S.L., *et al.*, 2004). The mean (SD) time-trade-off utility measure for all skin conditions was 0.943 (0.124), but ranged from 0.640 to 1.000 for specific skin conditions, depending on the disorder in question. For example, the mean (SD) utility for acne vulgaris was 0.938 (0.124); for dermatitis, 0.939 (0.098); for non-melanoma skin cancer, 0.976 (0.052); and for actinic keratosis, 0.981 (0.056).

## 3. Dermatologic Changes in Older People and Their Impact on Health

The aging process differs among individuals based on genetic variability, the toxicity of byproducts of metabolic processes, and the sufficiency of physiologic resources available for somatic maintenance and repair (Guinot C.G. *et al.*, 2002). Guinot and coauthors (Guinot C.G. *et al.*, 2002) identified four categories of factors that contribute to the skin aging process: 1) biological (genetically predetermined and unalterable); 2) environmental (e.g., damage from exposure to sunlight, pollutants, and/or nicotine); 3) mechanical (e.g., repetitive muscle movements such as squinting or frowning); and 4) miscellaneous (e.g., sleep patterns, dietary intake, comorbid conditions, and mental health and well-being).

**Figure 2:**
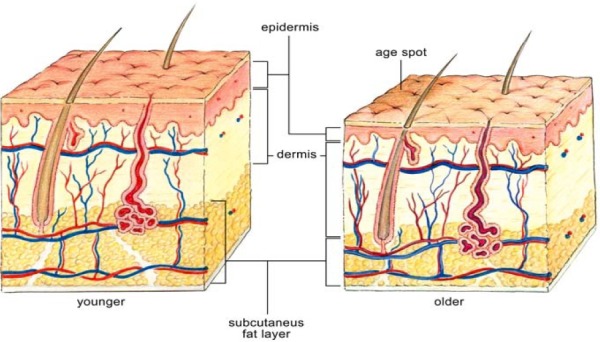
Changes of skin with age With kind permission from Informa HealthCare- Farage, M.A., Miller, K.W., Elsner, P. and Maibach, H.I. (2007). Structural characteristics of aging skin: a review. *Journal of Cutaneous and Ocular Toxicology, 26*, 343-357

Skin changes associated with aging are readily apparent (Table 1): thin, dry skin; age spots; wrinkles; prominent veins; etc. Such changes can be classified broadly as either age-related changes or as photoaging. Age-related skin changes are further classified as (1) functional or (2) structural. Functional changes include decreases in skin barrier function, mechanical protection, sensory perception, wound healing capability, immunologic responsiveness, thermoregulation, and vitamin D production. Structural changes lead to dryness, roughness, wrinkling, skin laxity, and decreased skin elasticity (http://www.merck.com/mkgr/mmg/sec15/ch122/ch122c.jsp). Structural changes emerge as the skin becomes progressively thinner during adulthood (Figure 2) (Farage M.A. *et al.*, 2007). Those changes include a reduction in the number of cells comprising the epidermis, changes in cellular shape, uneven pigmentation, reduced cutaneous immunity, reduced sebum production, and lower water content causing drier skin, even xerosis (Farage M.A. *et al.*, 2007).

Older adults are more likely to experience skin irritation or dermatologic disease than are younger adults. In fact, most persons over age 65 have two or more skin diseases/disorders that could require medical treatment (Kligman A.M. & Koblenzer C., 1997). Urinary and fecal incontinence, for example, can be common among older people; because aging skin is vulnerable, dermatologic complications associated with incontinence are frequent (Farage M.A. *et al.*, 2007). Untreated incontinence can lead to incontinence dermatitis, dermatological infections, intertrigo, vulvar folliculitis, and pruritus ani. Chronic incontinence can produce a continuing cycle of skin damage, irritation, and inflammation.

Skin cancer is also more prevalent among older persons. In recent years, public education about skin cancer prevention has raised awareness about strategies such as using sunscreen and reducing sun exposure. However, the Centers for Disease Control and Prevention report that eldely people have greater morbidity and mortality from skin cancer: men over age 65 account for 22% of incident cases of malignant melanoma among men, and women over age 65 account for 14% of incident melanomas among women each year (Counseling to prevent skin cancer, 2008).

### Gaps in Existing Health-Related Quality of Life Measures

Older adults experience a number of skin diseases and disorders that affect quality of life substantially. In the last two decades, a number of HRQoL measures have been developed for use in general dermatology patients or patients with specific dermatologic conditions to assess the effects of treatment and disease progression, perceptions of well-being, and the value that patients place on their dermatologic state of health. Some instruments have been validated and further refined over time, while others are in earlier stages of development. Opportunity thus exists for developing and validating HRQoL specifically for dermatological conditions most pertinent to older patients. Such instruments should cover aspects of HRQoL unique to, or at least more common to, elderly patients with dermatologic disorders, and should be validated in those populations.
